# IRF7 orchestrates maladaptive smooth muscle cell phenotype switching in atherosclerosis

**DOI:** 10.1093/pcmedi/pbaf039

**Published:** 2025-12-27

**Authors:** Rundong Cai, Xin Chen, Hongxia Zhang, Qi Wang, Wanrong Xie, Xinghua Pan, Chun Liang, Haiying Zhu

**Affiliations:** Department of Cardiology, Changzheng Hospital, Naval Medical University (Second Military Medical University), Shanghai 611230, China; Department of Cell Biology, College of Basic Medical Science, Naval Medical University (Second Military Medical University), Shanghai 571623, China; Department of Cell Biology, College of Basic Medical Science, Naval Medical University (Second Military Medical University), Shanghai 571623, China; Department of Cell Biology, College of Basic Medical Science, Naval Medical University (Second Military Medical University), Shanghai 571623, China; Department of Cell Biology, College of Basic Medical Science, Naval Medical University (Second Military Medical University), Shanghai 571623, China; Viterbi School of Enginnering, University of Southern California, Los Angeles, CA 91010, USA; Precision Regenerative Medicine Research Centre, Medical Science Division, Macau University of Science and Technology, Macao 999078, China; Department of Biochemistry and Molecular Biology, School of Basic Medical Sciences, and Guangdong Provincial Key Laboratory of Single-cell and Extracellular Vesicles, Southern Medical University, Guangzhou 510515, China; Department of Cardiology, Changzheng Hospital, Naval Medical University (Second Military Medical University), Shanghai 611230, China; Department of Cell Biology, College of Basic Medical Science, Naval Medical University (Second Military Medical University), Shanghai 571623, China

**Keywords:** atherosclerosis, vascular smooth muscle cell, IRF7, phenotype switching, single-cell RNA sequencing, transdifferentiation, inflammation, macrophage-like cells

## Abstract

**Background:**

Smooth muscle cells (SMCs) exhibit remarkable plasticity, undergoing extensive phenotypic switching to generate a highly heterogeneous population within atherosclerotic plaques. While recent studies have highlighted the contribution of SMC-derived macrophage-like cells to plaque inflammation, the specific molecular drivers governing the transition to these pathogenic states remain poorly understood.

**Methods:**

Here, we re-analyzed single-cell RNA sequencing data from lineage-traced mice to dissect SMC heterogeneity during atherogenesis. Trajectory analysis revealed that SMCs transdifferentiate into a distinct pro-inflammatory macrophage-like subpopulation (macrophage 4) via an intermediate “stem–endothelial–monocyte" cell state. Integrated gene regulatory network inference and *in silico* perturbation modeling identified interferon regulatory factor 7 (IRF7) as a master transcriptional regulator orchestrating this specific pathogenic transition.

**Results:**

Clinically, IRF7 expression was significantly upregulated in unstable and advanced human atherosclerotic plaques, correlating strongly with inflammatory macrophage burden. *In vivo, ApoE*^−/−^ mice challenged with a high-fat diet exhibited robust upregulation of IRF7 in aortic plaques, which co-localized with macrophage markers. Crucially, SMC-specific knockdown of *Irf7* using an AAV-SM22α-shIRF7 vector significantly attenuated atherosclerotic plaque progression, reduced necrotic core formation, and enhanced fibrous cap stability. Mechanistically, *Irf7* silencing preserved the contractile SMC phenotype and inhibited the accumulation of pro-inflammatory SMC-derived macrophage-like cells within the lesion.

**Conclusions:**

These findings identify IRF7 as a critical checkpoint in maladaptive SMC phenotype switching. We demonstrate that IRF7 drives the transdifferentiation of SMCs into a pro-inflammatory macrophage-like state, thereby fueling plaque instability. Consequently, therapeutic strategies capable of inhibiting IRF7-mediated SMC plasticity may prove effective in stabilizing vulnerable atherosclerotic plaques.

## Graphical Abstract

**Graphical Abstract:**
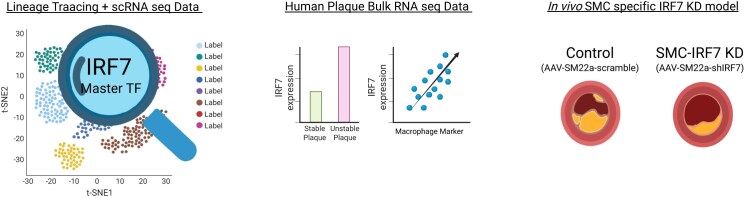
Single-cell RNA sequencing identifies the trajectory of SMC transdifferentiation into a pro-inflammatory macrophage-like state with IRF7 as the master regulator of this pathogenic transition. In human plaques, high IRF7 expression correlates with plaque instability. *In vivo* SMC-specific knockdown of *Irf7* inhibits the maladaptive phenotypic switching and stabilizes the atherosclerotic plaque.

## Introduction

Atherosclerosis serves as the fundamental pathological basis for ischemic heart disease and stroke, collectively remaining the leading causes of morbidity and mortality worldwide. Historically, the pathology of atherosclerosis was defined primarily by the formation of atheroma resulting from the excessive accumulation of lipids. However, over the past few decades, it has become clear that atherosclerosis is not simply a by-product of hypercholesterolemia, but a chronic inflammatory disease involving the participation of endothelial cells (ECs), smooth muscle cells (SMCs), and immune cells such as macrophages [1–3].

While immune cells were once considered the primary drivers of plaque progression, recent studies have revealed that SMCs are a major source of plaque cells [4]. The contribution of SMCs to atherosclerosis presents a significant biological paradox. On one hand, the migration of SMCs and their subsequent production of extracellular matrix are critical for forming the fibrous cap, the primary barrier against thrombotic rupture [5]. On the other hand, recent studies combining single-cell RNA sequencing (scRNA-seq) and lineage tracing technology reveal that this reparative response is highly heterogeneous [6]. A significant subset of these cells bypasses the stabilizing myofibroblast state, instead acquiring a degradative, synthetic phenotype that exacerbates inflammation [7, 8]. This duality highlights that SMCs are not merely a uniform population, but a mosaic of distinct subsets with opposing effects on plaque stability.

Despite the pivotal role of SMC plasticity, the specific transcriptional regulators that tip this balance toward a pathogenic phenotype remain largely undefined. In this study, utilizing scRNA-seq data, bulk RNA-seq data, and *in vivo* experimental models, we identify interferon regulatory factor 7 (IRF7) as a critical factor promoting SMC phenotype switching and driving the progression of atherosclerosis.

IRF7 is a transcription factor known to orchestrate immune responses in cancer, autoimmunity, and viral infections [9, 10]. Emerging evidence highlights a complex, cell-type-specific role for IRF7 in vascular pathology. In the context of vascular injury and remodeling, IRF7 functions as a protective factor within SMCs. Huang *et al*. demonstrated that IRF7 expression is downregulated following carotid artery injury, and its restoration inhibits neointima formation by suppressing SMC proliferation through the inhibition of the activating transcription factor 3 (ATF3) – proliferating cell nuclear antigen (PCNA) signaling axis [11]. Similarly, in pulmonary hypertension, IRF7 has been identified as a critical checkpoint that prevents pulmonary vascular remodeling; its overexpression attenuates pulmonary artery smooth muscle cell proliferation and inflammation, again by antagonizing ATF3-mediated signaling [12]. Conversely, in the context of diabetic atherosclerosis, IRF7 appears to play a detrimental role within the immune compartment. Senatus *et al*. reported that receptor for advanced glycation end products (RAGE) signaling upregulates IRF7 in macrophages, which in turn promotes inflammation and suppresses cholesterol efflux, thereby impairing plaque regression [13]. However, despite these insights into macrophage function and mechanical injury models, the specific contribution of SMC-derived IRF7 to the development and stability of lipid-driven atherosclerotic plaques remains unknown.

In this study, we utilized scRNA-seq combined with *in vivo* experimental models to dissect this heterogeneity. We identify IRF7 as a potential but critical factor promoting SMC phenotype switching and driving the progression of atherosclerosis.

## Methods

### Analysis of sc- and bulk RNA-seq data

The mouse scRNA-seq data utilized in this study were obtained from the Gene Expression Omnibus (GEO) database under accession number GSE155513, originating from the study by Pan *et al*. [14]. To investigate the phenotypic heterogeneity and transdifferentiation of SMCs during atherogenesis, the study employed a specific SMC-lineage-tracing mouse model generated by crossing ROSA26^ZsGreen1/+^ reporter mice with Myh11-CreER^T2^ mice. These lineage-tracing mice were subsequently crossed onto an Ldlr^−/−^ background and fed a Western diet (WD) to induce atherosclerosis.

Arterial tissues, including the ascending aorta, brachiocephalic artery, and thoracic aorta, were harvested at four distinct time points (after 0, 8, 16, and 26 weeks of WD feeding) to capture the temporal dynamics of plaque progression. To distinguish SMC-derived cells from other cell types, arterial tissues were digested into single-cell suspensions and subjected to fluorescence-activated cell sorting (FACS). This strategy separated cells into ZsGreen1-positive (SMC-lineage) and ZsGreen1-negative (non-SMC lineage) populations, which were then independently processed for scRNA-seq using the 10x Genomics Chromium platform.

The human carotid atherosclerosis scRNA-seq data utilized in this study were obtained from the GEO database under accession number GSE253903, originating from the study by Bashore *et al*. [6]. To investigate the clinical relevance of SMC phenotype switching and inflammatory burden, the dataset comprised carotid endarterectomy specimens collected from a cohort of patients clinically stratified into symptomatic and asymptomatic groups.

Human bulk RNA-seq data was used to evaluate the clinical relevance of IRF7 expression in different pathological stages and phenotypes of human atherosclerosis. Three independent bulk RNA-seq datasets were retrieved from the GEO database. The dataset GSE28829 was analyzed to compare IRF7 expression levels between stable and unstable atherosclerotic plaques [15]. The GSE163154 dataset was utilized to assess differential expression in plaques characterized by intraplaque hemorrhage (IPH) versus non-IPH lesions [16]. Additionally, the GSE120521 dataset was employed to examine IRF7 expression differences between early-stage and advanced atherosclerotic lesions [17]. For detailed bioinformation methods, see the online supplementary material.

### Animals and ethics statement

All animal experiments were conducted in accordance with the guidelines for the Care and Use of Laboratory Animals and were approved by the Ethics Committee of Second Military Medical University (No. 2024SL197). Male *ApoE* knockout (KO) *ApoE*^-/-^ mice on a C57BL/6J background (GemPharmatech Co., Ltd) were used for all experiments. Mice were housed in a specific pathogen-free facility under a 12-h light/dark cycle with *ad libitum* access to food and water.

### Evaluation of IRF7 expression in atherosclerosis

To investigate the expression pattern of IRF7 during atherogenesis, 12 *ApoE*^-/-^ mice were randomly assigned to two groups (*n* = 6 per group): a normal diet (ND) group fed a standard chow diet, and a high-fat diet (HFD) group fed a WD (Research Diets) to induce atherosclerosis.

### SMC-specific IRF7 knockdown mouse model

To determine the specific role of SMC-derived IRF7 in atherosclerosis, an adeno-associated virus (AAV) system was employed to knock down *Irf7* expression specifically in SMCs. AAV2/9 vectors carrying a short hairpin RNA (shRNA) targeting *Irf7* (AAV2/9-SM22α-shIRF7) or a scramble control sequence (AAV2/9-SM22α-scramble) under the control of the SMC-specific SM22α promoter were constructed (Anzhen Bio). A total of 24 male *ApoE*^-/-^ mice were randomly divided into two groups (*n* = 12 per group): the control group and the *Irf7* knockdown (KD) group. At 8 weeks of age, mice received a tail vein injection of the respective AAV vectors (5×10^11^ genome copies/mouse). Following a 1-week recovery period (at 9 weeks of age), all mice were challenged with a HFD for 20 weeks to promote atherosclerotic plaque formation.

### Tissue collection and histological analysis

To enable both morphological and molecular analyses from the same animal, the aorta was divided anatomically. The proximal portion, comprising the ascending aorta and the aortic arch, was fixed in 4% paraformaldehyde and processed for en face oil red O staining to quantify the gross atherosclerotic lesion area. The descending thoracic and abdominal aorta was immediately snap-frozen in liquid nitrogen and stored at −80°C for subsequent protein extraction and western blot analysis. Additionally, the upper portion of the heart containing the aortic root was embedded in optimal cutting temperature (OCT) compound (Sakura) or paraffin. Serial cryosections of the aortic sinus were prepared for histological assessment. These sections were subjected to hematoxylin and eosin (H&E) staining for plaque morphology, oil red O staining for lipid accumulation, and Masson’s trichrome and sirius red staining to evaluate collagen content.

### Western blot

Proteins were extracted from thoracic and abdominal aortic tissues using radioimmunoprecipitation assay (RIPA) lysis buffer containing protease and phosphatase inhibitors. Equal amounts of protein were separated by sodium dodecyl sulfate–polyacrylamide gel electrophoresis (SDS-PAGE) and transferred onto polyvinylidene difluoride (PVDF) membranes. After blocking with 5% non-fat milk, membranes were incubated overnight at 4°C with primary antibodies against IRF7, α-SMA, CD68, and glyceraldehyde-3-phosphate dehydrogenase (GAPDH). Membranes were then washed and incubated with horseradish peroxidase (HRP)-conjugated secondary antibodies for 1 h. Protein bands were visualized using an ECL detection system and quantified using ImageJ software. All the antibodies used and their dilution ratios are listed in supplementary Table 1, see online supplementary material.

### Multiplex immunohistochemistry

To simultaneously visualize the spatial distribution of IRF7, CD68, and alpha-smooth muscle actin (α-SMA) on the same tissue section, multiplex immunohistochemistry (mIHC) was performed using tyramide signal amplification (TSA) technology (RecordBio). Following deparaffinization, antigen retrieval (pH 9.0, Tris-EDTA for 1 h) and blocking (Beyotime), tissue sections were subjected to three sequential rounds of staining. In the first cycle, sections were incubated with anti-IRF7 antibody overnight at 4°C followed by HRP-conjugated secondary antibody and detection with TSA-570. The antibody–HRP complex was stripped via microwave heat treatment. In the second cycle, sections were incubated with anti-CD68 antibody and detected with TSA-520. In the third cycle, anti-α-SMA antibody was applied and detected with TSA-690. Finally, nuclei were counterstained with Hoechst (Thermo Fisher Scientific) and slides were mounted with antifade medium (Thermo Fisher Scientific). All the antibodies used and their dilution ratios are listed in supplementary Table 2, see online supplementary material).

### IHC and immunofluorescence staining

To characterize plaque composition, IHC staining was performed on paraffin-embedded aortic root sections. Sections were deparaffinized in xylene and rehydrated through a graded ethanol series. Antigen retrieval was achieved by heating slides in citrate buffer (pH 6.0) for 1 h. Endogenous peroxidase activity was quenched with 3% hydrogen peroxide, and non-specific binding was blocked with quick-blocking buffer (Beyotime). Sections were incubated overnight at 4°C with primary antibodies against CD68 to identify macrophages and α-SMA to identify SMCs. Detection was performed using a HRP-conjugated secondary antibody polymer system followed by incubation with 3,3'-diaminobenzidine (DAB) substrate (Servicebio), yielding a brown reaction product. Nuclei were counterstained with hematoxylin, and images were captured using a light microscope. For standard assessment of cellular abundance, sections were incubated overnight with primary antibodies against CD68 and α-SMA, followed by species-specific fluorophore-conjugated secondary antibodies for 1 h at room temperature. All the antibodies used and their dilution ratios are listed in supplementary Table 2.

### Statistical analysis

All statistical analyses were performed using GraphPad Prism software (version 9.0). Data are presented as the mean ± standard error of the mean (SEM). The normality of data distribution was assessed using the Shapiro–Wilk test. For comparisons between two experimental groups (e.g. ND vs. HFD; control vs. *Irf7* KD), statistical significance was determined using an unpaired, two-tailed Student’s t-test for normally distributed data or a Mann–Whitney U test for non-normally distributed data. For comparisons involving more than two groups, one-way analysis of variance (ANOVA) was used, followed by Tukey’s *post hoc* test for multiple comparisons. A *P*-value < 0.05 was considered statistically significant.

## Results

### Single-cell transcriptomic analysis reveals SMC transdifferentiation into distinct macrophage-like states during atherogenesis

**Figure 1:**
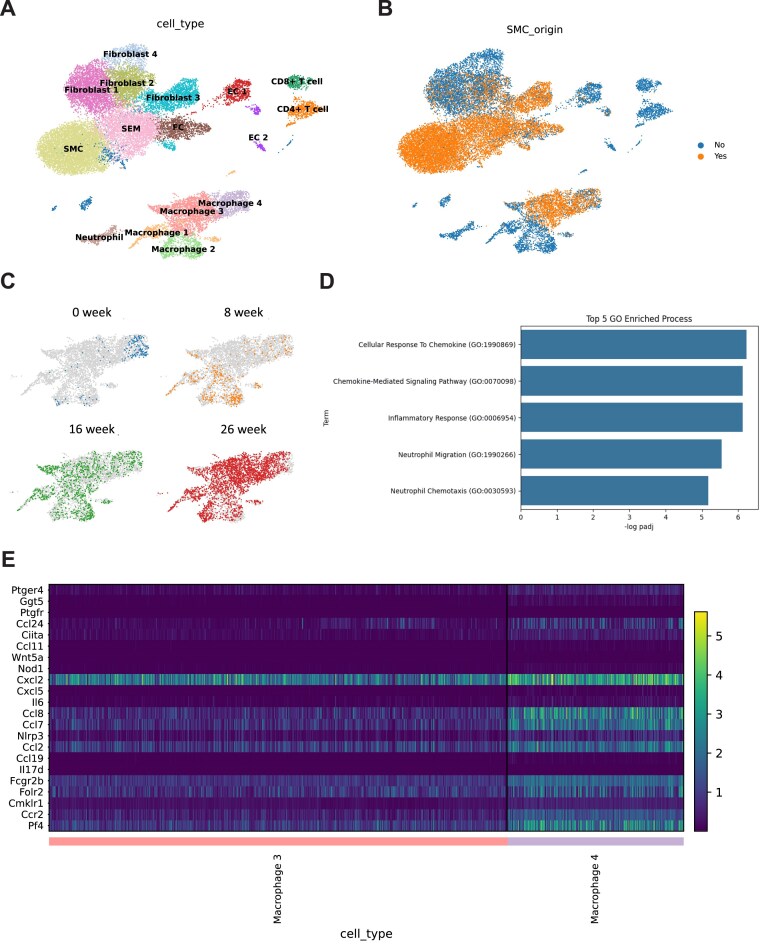
Single-cell transcriptomic analysis reveals SMC transdifferentiation into distinct macrophage-like states during atherogenesis. (A) Uniform manifold approximation and projection visualization of 16 distinct cell clusters identified from the aortic single-cell dataset (GSE155513). Clusters were annotated based on canonical marker gene expression. (B) Lineage tracking of SMCs. Cells of SMC origin (ZsGreen1+) are highlighted in yellow. Note the significant contribution of SMC-lineage cells to macrophage clusters 3 and 4. (C) Temporal evolution of SMC-lineage cells across disease progression (0, 8, 16, and 26 weeks of WD). The transition from contractile SMCs to macrophage-like states is prominent in advanced stages (16 and 26 weeks). (D) Gene ontology (GO) enrichment analysis comparing the biological processes between SMC-derived macrophage 4 and macrophage 3. The top five enriched terms in macrophage 4 are associated with inflammatory responses. (E) Heatmap showing the relative expression of genes involved in the “inflammatory response” GO term. Macrophage 4 exhibits a distinct pro-inflammatory transcriptional profile compared to macrophage 3.

To dissect the cellular heterogeneity and lineage trajectories within atherosclerotic lesions, we utilized the scRNA-seq dataset from a study published in Circulation in 2020 by Pan *et al*. [14]. This dataset profiled aortic cells derived from lineage-traced *ROSA26^ZsGreen1/+^; Ldlr^−/-;^ Myh11-CreER^T2^* mice fed a WD for 0, 8, 16, and 26 weeks to capture the dynamic progression of plaque development. Following rigorous quality control and doublet removal, we re-analyzed the data and identified 16 distinct cell clusters via unsupervised clustering, which were annotated based on the expression of canonical marker genes (Fig. 1A). The cell type identities were further confirmed by analyzing the top differentially expressed genes for each cluster (supplementary Fig. 1A, see online supplementary material).

We next utilized the lineage-tracing information embedded in the dataset to distinguish cells of SMC origin (ZsGreen1+) from those of non-SMC origin. While the majority of SMC-lineage cells clustered within the canonical SMC and fibroblast populations, a significant fraction mapped to clusters identified as macrophages (Fig. 1B). Specifically, among the four macrophage clusters identified (macrophage 1–4), macrophage 3 and macrophage 4 were predominantly of SMC origin, indicating extensive transdifferentiation of SMCs into macrophage-like phenotypes. Temporal analysis revealed that this phenotypic switching is a progressive process; while SMC identity was largely preserved at early time points (weeks 0 and 8), a profound shift toward these macrophage-like states was observed at advanced stages of atherosclerosis (weeks 16 and 26) (Fig. 1C).

Given that both macrophage 3 and macrophage 4 originated from SMCs yet formed distinct clusters, we hypothesized that they represent functionally distinct states. To elucidate the molecular differences between them, we performed differential expression followed by gene ontology (GO) enrichment analysis. The results revealed a striking functional divergence: the top five enriched biological pathways in macrophage 4 were exclusively associated with inflammatory responses, including “inflammatory response,” “cytokine production,” and “response to bacterium” (Fig. 1D).

To further validate this pro-inflammatory phenotype, we examined the expression profiles of specific inflammation-related genes. A heatmap of genes associated with the “inflammatory response” GO term demonstrated that macrophage 4 exhibited significantly higher expression of pro-inflammatory mediators compared to macrophage 3 (Fig. 1E). This observation was corroborated by feature plots showing the intense expression of typical pro-inflammatory markers (e.g. Ccl2, Cxcl2, Nlrp3) specifically within the macrophage 4 cluster (supplementary Fig. 1B–G). Collectively, these analyses demonstrate that during advanced atherosclerosis, a subpopulation of SMCs transdifferentiates into a specific, highly pro-inflammatory macrophage-like state (macrophage 4), distinct from other SMC-derived populations.

### Integrated regulatory network analysis identifies IRF7 as a key driver of the pro-inflammatory SMC–macrophage transition

**Figure 2:**
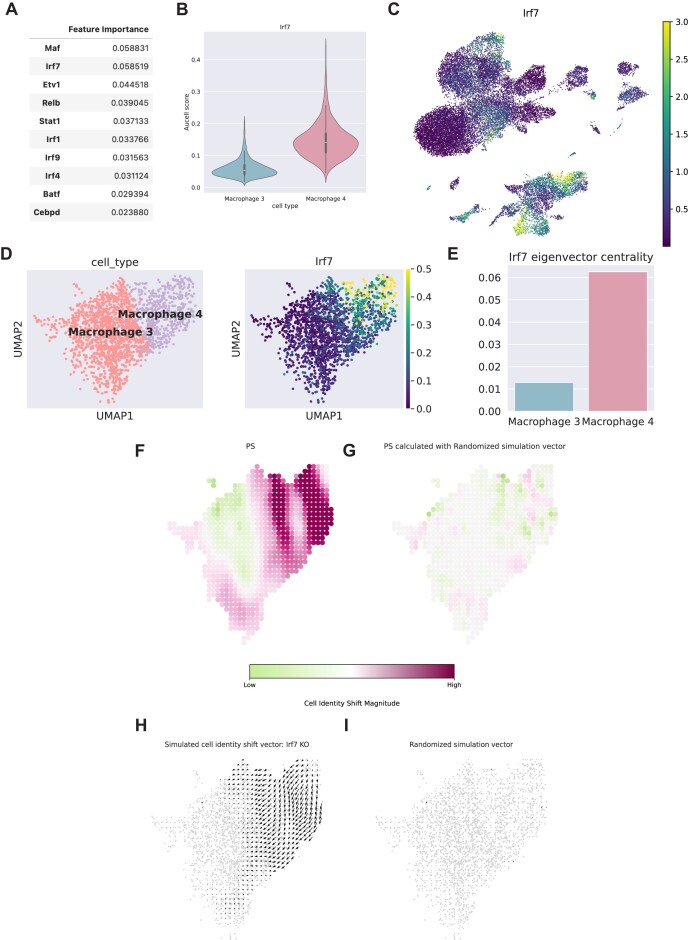
Integrated regulatory network analysis identifies IRF7 as a key driver of the pro-inflammatory SMC–macrophage transition. (A) Ranking of top TFs distinguishing macrophage 4 from 3, identified by random forest classification based on SCENIC regulon activity. IRF7 is identified as a top-ranking candidate. (B) Violin plot showing the distribution of IRF7 regulon activity (AUCell score) in macrophage 3 vs. macrophage 4 clusters. (C) Feature plot visualizing the spatial distribution of *Irf7* gene expression on uniform manifold approximation and projection (UMAP) embedding. (D) CellOracle visualization of imputed *Irf7* gene expression within the SMC-derived macrophage sub-lineage. (E) Network centrality analysis. Bar chart showing the eigenvector centrality score of IRF7 in the gene regulatory networks of macrophage 3 and macrophage 4, indicating its higher regulatory influence in macrophage 4. (F–I) *In silico* perturbation analysis using CellOracle. (F) Simulation of *Irf7* KO showing the higher perturbation score (PS) (magnitude of cell identity shift, maroon color indicates high shift) compared a to randomized control (G). (H) Vector field analysis showing the predicted developmental trajectory upon *Irf7* KO, where arrows indicate a shift away from the macrophage 4 phenotype toward macrophage 3, compared to control (I).

To unravel the molecular mechanisms governing the bifurcation of SMCs into distinct macrophage-like states—specifically the pro-inflammatory macrophage 4 vs. the less inflammatory macrophage 3—we sought to identify key transcription factors (TFs) directing these lineage decisions. We employed the pySCENIC pipeline to reconstruct the gene regulatory network and quantify regulon activity at the single-cell level [18]. By applying a random forest classification algorithm to the regulon activity matrix, we ranked TFs based on their importance in distinguishing macrophage 4 from macrophage 3. This analysis identified IRF7 as a top-ranking candidate driver (ranking second among all identified TFs) (Fig. 2A). Consistent with this ranking, the regulon activity (AUCell score) of IRF7 was significantly elevated in the pro-inflammatory macrophage 4 cluster compared to macrophage 3 (Fig. 2B), a pattern mirrored by the global gene expression distribution (Fig. 2C).

To further validate the regulatory hierarchy of IRF7, we utilized CellOracle to model cell-type-specific gene regulatory networks [19]. As a prerequisite for network modeling, we visualized the imputed gene expression within the specific macrophage sub-lineage, which confirmed the distinct enrichment of *Irf7* in the macrophage 4 population (Fig. 2D). We then calculated eigenvector centrality, a metric quantifying a gene’s influence within the inferred network. Strikingly, IRF7 exhibited a high centrality score specifically within the macrophage 4 network, whereas its centrality was negligible in macrophage 3 (Fig. 2E). This indicates that IRF7 functions as a critical regulatory hub essential for establishing or maintaining macrophage 4 identity.

Finally, to predict the functional consequence of IRF7 loss on cell fate, we performed *in silico* perturbation analysis using CellOracle. We simulated a specific KO of *Irf7* within the inferred gene regulatory network and modeled the resulting shift in cellular identity. The perturbation simulation map revealed a strong shift magnitude (indicated by deep maroon color) specifically localized to the macrophage 4 cluster (Fig. 2F), in contrast to the randomized control (Fig. 2G). Vector field analysis of the differentiation trajectory demonstrated that *Irf7* KO fundamentally altered the developmental flow: rather than maintaining the macrophage 4 state, the velocity vectors reversed direction, pointing toward the less inflammatory macrophage 3 phenotype (Fig. 2H and I). Collectively, these computational models suggest that IRF7 is a requisite factor for driving SMC phenotypic switching toward the pro-inflammatory macrophage-like state.

### Elevated IRF7 expression correlates with plaque instability and inflammatory macrophage accumulation in human atherosclerosis

**Figure 3:**
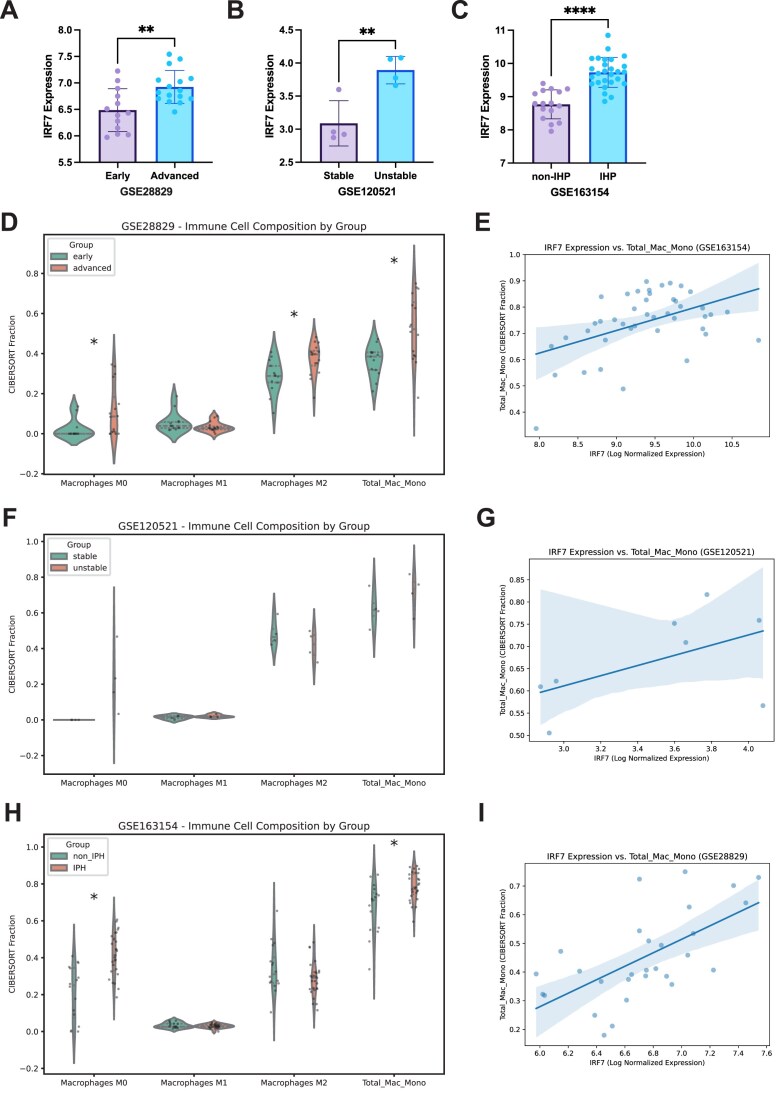
Elevated IRF7 expression correlates with plaque instability and inflammatory macrophage accumulation in human atherosclerosis. (A–C) Analysis of IRF7 mRNA expression in human atherosclerotic plaques from three independent GEO datasets. (A) Unstable vs. stable plaques (GSE28829). (B) Advanced vs. early plaques (GSE120521). (C) IPH vs. non-IPH plaques (GSE163154). (D, F, H) CIBERSORT immune deconvolution analysis showing the relative fractions of macrophages and monocytes in the respective datasets. (E, G, I) Spearman correlation analysis between IRF7 expression levels and total macrophage content in human plaques. Data are presented as mean ± SEM. *P < 0.05, **P < 0.01, ***P < 0.001, ****P < 0.0001.

To validate the clinical relevance of our findings, we analyzed IRF7 expression in three independent human bulk RNA-seq datasets representing different stages of atherosclerotic plaque progression. Across all cohorts, IRF7 expression was significantly upregulated in complex, vulnerable lesions compared to stable or early-stage plaques. Specifically, IRF7 levels were elevated in advanced vs. early-stage atheroma (GSE28829; Fig. 3A), in unstable vs. stable plaques(GSE120521; Fig. 3B), and in lesions with IPH vs. non-IPH lesions (GSE163154; Fig. 3C). These data indicate that IRF7 expression scales with disease severity and plaque instability in humans.

To determine if this upregulation reflects the accumulation of the specific SMC-derived macrophage population identified in our mouse model, we generated a “macrophage 4” gene signature based on the top 100 differentially expressed genes defined in our scRNA-seq analysis. Single-sample gene set enrichment analysis (ssGSEA) revealed that this pro-inflammatory macrophage 4 signature was significantly enriched in severe atherosclerotic lesions. (supplementary Fig. 2B, D, F, see online supplementary material), suggesting that the expansion of this specific cell state is a conserved feature of advanced human atherosclerosis.

Furthermore, we characterized the immune landscape of these plaques using CIBERSORT deconvolution. Consistent with the inflammatory nature of advanced disease, severe plaques (unstable, IPH, advanced) exhibited a significantly higher proportion of total macrophages and monocytes compared to their stable counterparts (Fig. 3D, F, H; supplementary Fig. 2A, C, E). Notably, Spearman correlation analysis demonstrated a robust positive correlation between IRF7 expression and total macrophage content (Fig. 3E, G, I). While the specific polarization status (M1 vs. M2) showed variable correlations with IRF7 (supplementary Fig. 2G–H), the consistent association with total macrophage burden and the specific macrophage 4 signature strongly supports the hypothesis that IRF7 drives the accumulation of pro-inflammatory, SMC-derived macrophage-like cells in human atherosclerotic plaques.

### IRF7 expression is upregulated and localizes to macrophage-rich regions in atherosclerotic plaques

To investigate the expression dynamics of IRF7 during atherogenesis *in vivo*, we established a murine model of atherosclerosis using *ApoE*^*−/−*^ mice challenged with a HFD or a ND. As expected, en face oil red O staining revealed extensive atherosclerotic lesion formation in the aortas of the HFD group, whereas lesions were negligible in the ND group (supplementary Fig. 3A, see online supplementary material). This was corroborated by cross-sectional analysis of the aortic root, where HFD feeding resulted in significant plaque burden and lipid accumulation as visualized by H&E and oil red O staining (supplementary Fig. 3B, C).

**Figure 4:**
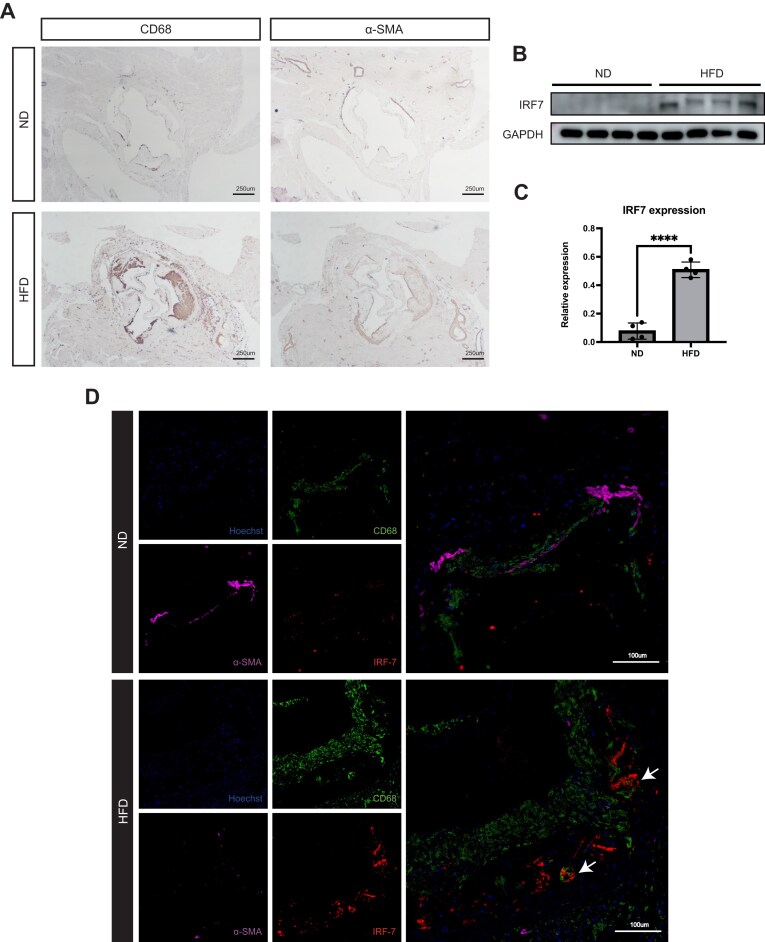
IRF7 expression is upregulated and localizes to macrophage-rich regions in atherosclerotic plaques. (A) Representative IHC staining of CD68 (macrophages) and α-SMA (SMCs) in aortic root sections from *ApoE^−/−^* mice fed a ND or HFD. Arrows indicate SMC migration to the sub-endothelial space. (B) Representative western blot analysis of IRF7 protein expression in aortic tissues from ND and HFD groups. (C) Quantification of IRF7 protein levels normalized to GAPDH. (D) mIHC staining of aortic root sections. IRF7 (red), CD68 (green), and α-SMA (magenta) are visualized. Nuclei are counterstained with Hoechst (blue). Note the significant upregulation of IRF7 in HFD plaques and its co-localization with CD68. Arrows show the co-localization of CD68 and IRF7. Data are presented as mean ± SEM. ****P < 0.0001.

Immunohistochemical characterization of the plaque composition confirmed a robust inflammatory response, characterized by significantly elevated expression of the macrophage marker CD68 in the HFD group compared to controls (Fig. 4A). Consistent with the typical pathology of advanced lesions, α-SMA-positive cells were observed migrating from the tunica media into the sub-endothelial space, contributing to the formation of the fibrous cap (Fig. 4A).

Concomitant with this plaque development, western blot analysis of aortic tissues demonstrated a significant upregulation of IRF7 protein levels in the HFD group compared to the ND control (Fig. 4B and C). To spatially resolve this expression within the plaque architecture, we utilized multiplex mIHC. While IRF7 signal was minimal in the healthy vessel wall (ND), it was robustly expressed within the atherosclerotic plaques of HFD-fed mice. Notably, IRF7 immunoreactivity exhibited partial colocalization with CD68 (Fig. 4D), supporting our single-cell findings that IRF7 is enriched in cell populations acquiring macrophage-like traits during atherogenesis.

### IRF7 drives the pro-inflammatory state of SMC-derived macrophage-like cells in symptomatic human atherosclerosis

**Figure 5:**
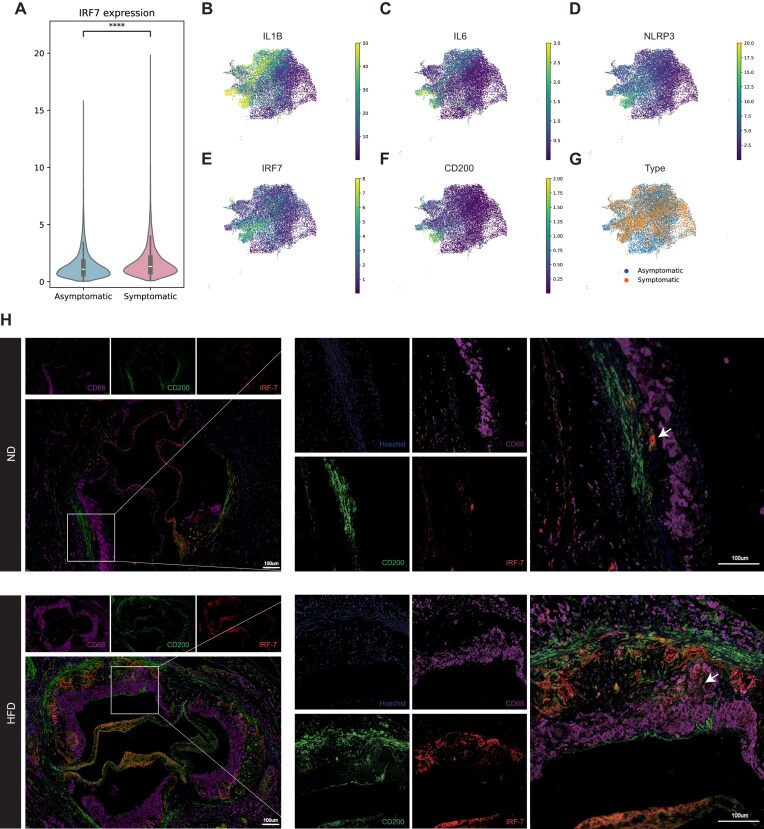
IRF7 is enriched in pro-inflammatory SMC-derived macrophage-like cells in unstable human plaques and identifies the phenotypic transition zone *in vivo*. (A) Violin plot showing the differential expression of IRF7 in total macrophages isolated from asymptomatic (*n* = 6) vs. symptomatic (*n* = 6) human carotid plaques (dataset: Bashore *et al*., 2024). IRF7 is significantly upregulated in symptomatic lesions. (B–D) Feature plots visualizing the expression of major pro-inflammatory cytokines (B) IL1B, (C) IL6, and (D) NLRP3 on uniform manifold approximation and projection embedding of human plaque macrophages. (E–F) Feature plots showing the spatial expression of (E) IRF7 and (F) the SMC-lineage marker CD200. Notably, the cluster located in the bottom-left region of the manifold exhibits a specific dual-positive CD200^+^/IRF7^high^ signature. (G) UMAP embedding colored by clinical status (Blue: Asymptomatic; Orange: Symptomatic). This pathogenic CD200^+^/IRF7^high^ subpopulation (bottom-left) is strikingly enriched in symptomatic patients, indicating a correlation with plaque instability. (H) Representative multiplex immunofluorescence staining of aortic root sections from ND and HFD *ApoE^−/−^* mice. Sections were stained for CD200 (SMC lineage marker), CD68 (macrophage marker), and IRF7. Arrows show the cells that were triple-positive for CD200, CD68, and IRF7. Data are presented as mean ± SEM. ****P < 0.0001.

To determine the clinical relevance of IRF7-mediated SMC transdifferentiation, we analyzed a high-dimensional single-cell multimodal dataset of human carotid atherosclerosis containing both symptomatic (*n* = 6) and asymptomatic (*n* = 6) plaques (Bashore et al., Arterioscler Thromb Vasc Bio 2024) [6]. We first isolated the total macrophage population based on the expression of canonical markers ITGAM, CD68, and CD14 (supplementary Fig. 4A–D, see online supplementary material). Strikingly, IRF7 expression was significantly upregulated in macrophages derived from symptomatic plaques compared to asymptomatic controls (Fig. 5A), suggesting a link between IRF7 activity and plaque instability.

A major challenge in human atherosclerosis research is distinguishing bone marrow-derived macrophages from SMC-derived macrophage-like cells. To address this, we leveraged CD200, which was recently identified as a robust, lineage-retained surface marker specific to human and murine SMCs, even after phenotypic switching (Bashore et al., Circulation 2024) [20]. We stratified the macrophage population based on inflammatory status and found that cells with high expression of major pro-inflammatory cytokines—IL1B, IL6, and NLRP3 (Fig. 5B–D)—strongly co-localized with high levels of both CD200 and IRF7 (Fig. 5E and F). This triple-positive signature identifies a specific subpopulation of SMC-derived macrophage-like cells that are inherently pro-inflammatory. Notably, these CD200^+^/IRF7^high^ cells were predominantly enriched in symptomatic patients (Fig. 5G), reinforcing the hypothesis that IRF7-driven SMC transdifferentiation contributes to the inflammatory milieu of unstable plaques.

### Spatial localization confirms IRF7 expression at the SMC–macrophage transition zone

To physically validate this transition *in vivo*, we performed mIHC staining for CD200, CD68, and IRF7 in aortic roots from ND and HFD *ApoE^−/−^* mice. In ND mice, the vessel wall consisted of contractile SMCs that were CD200^+^ but negative for IRF7 and CD68, confirming the baseline quiescence of these cells (Fig. 5H).

In contrast, HFD-fed mice exhibited complex plaques with distinct spatial architecture. We observed a CD200^+^ fibrous cap/media region (SMC-lineage) and a distinct CD68^+^ core region (macrophage-rich). Crucially, IRF7 expression was robustly induced in the HFD group and showed near-perfect co-localization with CD200 in the plaque area (Fig. 5H). Closer examination of the “transition zone”—the boundary interface between the SMC-rich cap and the macrophage-rich core—revealed a population of cells that were triple-positive for CD200, CD68, and IRF7 (Fig. 5H). The presence of these CD200^+^/CD68^+^ cells specifically in IRF7-rich regions provides direct histological evidence that IRF7 is actively expressed during the transdifferentiation of SMCs into macrophage-like cells *in situ*.

### SMC-specific KD of *Irf7* attenuates atherosclerotic plaque progression and enhances plaque stability

**Figure 6:**
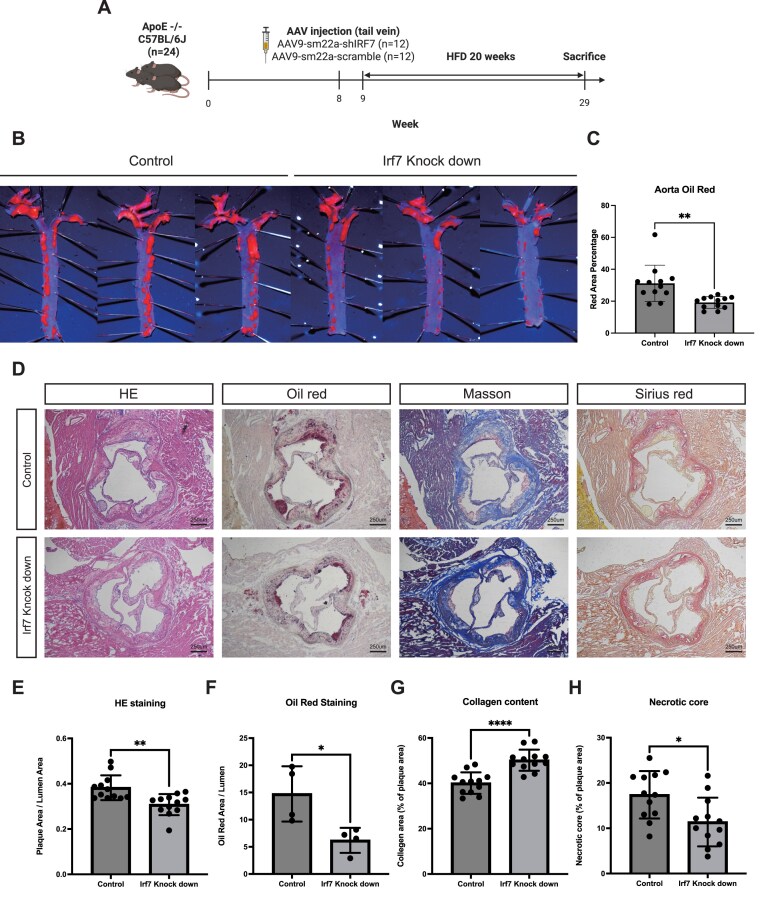
SMC-specific KD of Irf7 attenuates atherosclerotic plaque progression and enhances plaque stability. (A) Schematic diagram of the experimental design. ApoE^−/−^ mice were injected with AAV2/9-SM22α-shIRF7 (KD) or AAV2/9-SM22α-Scramble (control) and fed a WD for 20 weeks. (B) Representative en face oil red O staining of the aorta. (C) Quantification of the total lesion area percentage. (D) Representative histological staining of aortic root sections: H&E, oil red O, Masson’s trichrome, and sirius red. (E–H) Quantitative analysis of aortic root lesions: (E) plaque area, (F) lipid area (oil red O positive), (G) collagen content (Masson positive), and (H) necrotic core area. Data are presented as mean ± SEM. *P < 0.05, **P < 0.01, ***P < 0.001, ****P < 0.0001.

To definitively establish the functional role of SMC-derived IRF7 in atherogenesis, we employed an AAV serotype 2/9 system to specifically knock down *Irf7* in smooth muscle cells. We constructed a vector expressing a short hairpin RNA targeting *Irf7* (shIRF7) driven by the SMC-specific SM22α promoter. *ApoE^−/−^* mice were randomized into control (scramble) and *Irf7* KD groups, injected with the AAV vectors, and subsequently challenged with a WD for 20 weeks to induce atherosclerosis (Fig. 6A).

Gross morphological analysis via en face oil red O staining revealed a striking reduction in the total atherosclerotic lesion burden across the aorta in the *Irf7* KD group compared to controls (Fig. 6B and C). This observation was corroborated by histological assessment of the aortic root; H&E staining demonstrated a significant decrease in average plaque size in mice with SMC-specific *Irf7* silencing (Fig. 6D and E). Furthermore, cross-sectional oil red O staining indicated significantly reduced lipid accumulation within the lesions of the *Irf7* KD group (Fig. 6D and F).

Beyond plaque size, we evaluated the metrics of plaque stability. Masson’s trichrome staining revealed that plaques from *Irf7* KD mice contained significantly higher collagen content compared to controls, indicative of a thicker, more stable fibrous cap (Fig. 6D, G). Consistent with this stable phenotype, the area of the necrotic core was significantly reduced in the *Irf7* KD group (Fig. 6D, H).

Importantly, these atheroprotective effects were independent of systemic metabolic changes. We observed no significant differences in body weight, serum total cholesterol, triglycerides, low-density lipoprotein cholesterol (LDL-C), or high-density lipoprotein cholesterol (HDL-C) levels between the control and *Irf7* KD groups (supplementary Fig. 5A–E, see online supplementary material). Collectively, these *in vivo* findings highlight the profound impact of SMC-specific IRF7 on lesion development, identifying it as a pivotal driver of plaque progression and destabilization independent of systemic lipid metabolism.

### SMC-specific *Irf7* KD inhibits phenotypic switching and preserves plaque stability

**Figure 7:**
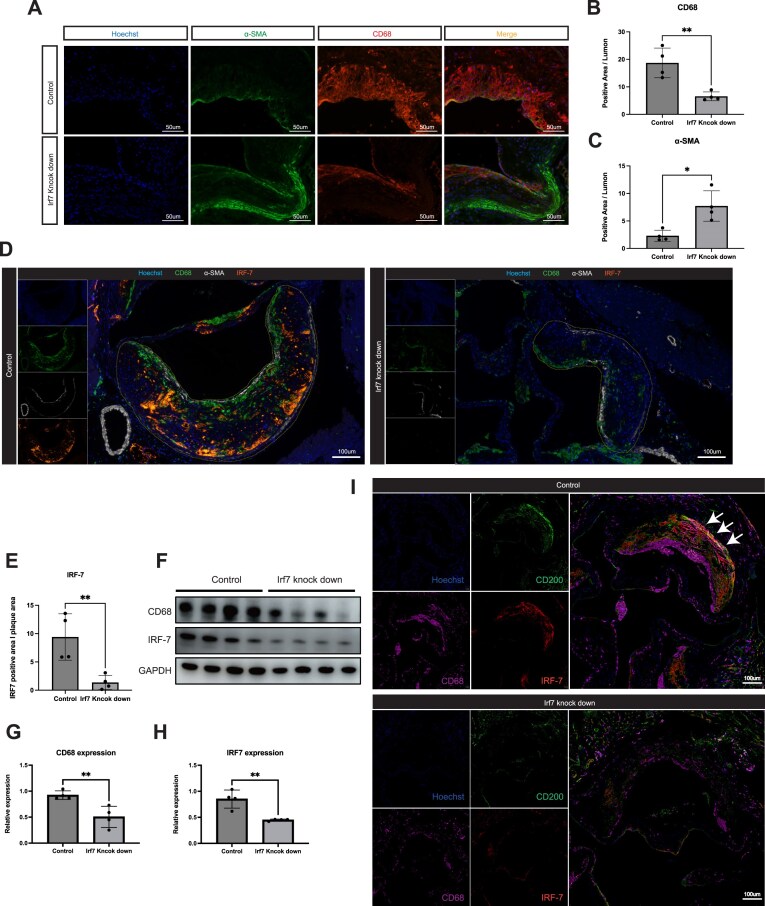
SMC-specific *Irf7* KD inhibits phenotypic switching and preserves plaque stability. (A) Representative IF staining of CD68 (red) and α-SMA (green) in aortic root sections from control and *Irf7* KD mice. Nuclei are stained with Hoechst (blue). (B and C) Quantification of the CD68-positive area (B) and α-SMA-positive area (C) within the plaque. (D) Representative mIHC staining validating the efficiency of *Irf7* KD in the plaque. (E) Quantification of the IRF7-positive area. (F) Representative western blot analysis of IRF7 and CD68 protein levels in aortic tissues from control and *Irf7* KD mice. (G and H) Quantification of CD68 (G) and IRF7 (H) protein levels normalized to GAPDH. (I) Representative multiplex immunofluorescence staining of CD200 (SMC lineage), CD68 (macrophage), and IRF7 in aortic roots from control and *Irf7* KD mice. Arrows show the cells that were triple-positive for CD200, CD68, and IRF7. Data are presented as mean ± SEM. *P < 0.05, **P < 0.01, ***P < 0.001.

To interrogate the cellular mechanisms underlying the atheroprotective effects of *Irf7* silencing, we performed IF staining to characterize the cellular composition of the plaques. In the control group, plaques were characterized by a large necrotic core and abundant CD68^+^ macrophage-like cells, with α-SMA expression largely confined to a thin fibrous cap at the shoulder regions. In striking contrast, plaques from the SMC-specific *Irf7* KD group exhibited a significantly preserved α-SMA^+^ smooth muscle cell population throughout the lesion (Fig. 7A). Quantitative analysis confirmed a significant reduction in the CD68^+^ area and a concomitant increase in the α-SMA^+^ area in the *Irf7* KD group compared to controls (Fig. 7B and C). These shifts in cellular composition are indicative of a more stable, less inflammatory plaque phenotype.

To validate the efficiency of the *Irf7* KD model and confirm the loss of IRF7 specifically within the plaque microenvironment, we utilized mIHC. While control plaques displayed high levels of IRF7 expression, the *Irf7* KD group showed negligible immunoreactivity, confirming effective silencing of the target protein (Fig. 7D). Quantification of the IRF7-positive area further substantiated this significant reduction (Fig. 7E).

Corroborating these findings at the protein level, western blot analysis of aortic lysates demonstrated rigorous suppression of IRF7 in the KD group (Fig. 7F). Importantly, this loss of IRF7 was accompanied by a significant downregulation of the macrophage marker CD68 (Fig. 7G and H).

To definitively distinguish whether the observed reduction in plaque macrophage burden was due to decreased monocyte infiltration or blocked SMC transdifferentiation, we employed the CD200 lineage-tracing strategy in our AAV-mediated *Irf7* KD model. In the control group, we consistently observed the large proportion of pathogenic triple-positive population (CD200^+^/CD68^+^/IRF7^+^) in plaque, confirming active maladaptive phenotype switching driven by endogenous IRF7 (Fig. 7I, top panel). Strikingly, SMC-specific silencing of *Irf7* almost completely abolished this triple-positive population. In the *Irf7* KD group, the majority of CD200^+^ cells retained a non-macrophage phenotype (CD68^−^). This result provides direct *in vivo* evidence that IRF7 expression is functionally required for SMCs to shed their contractile identity and acquire macrophage-like traits (Fig. 7I, bottom panel). By blocking this IRF7-dependent trajectory, we effectively preserved the SMC lineage identity and prevented the accumulation of SMC-derived inflammatory cells within the lesion.

## Discussion

**Figure 8:**
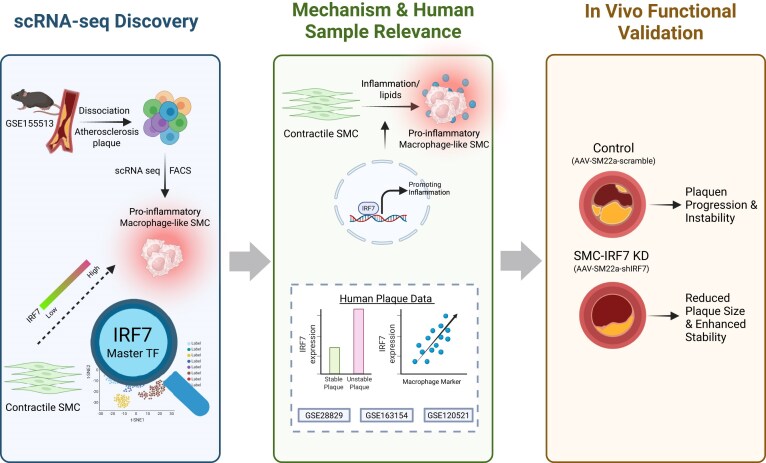
Schematic illustration summarizing the study. Left: (1) scRNA-seq identifies a trajectory of SMC transdifferentiation into a pro-inflammatory macrophage-like state (macrophage 4) via an intermediate “stem–endothelial–monocyte” cell state. Middle: (2) integrated network analysis identifies IRF7 as the master regulator of this pathogenic transition. In human plaques, high IRF7 correlates with instability. Right: (3) *in vivo* SMC-specific KD of *Irf7* inhibits this phenotypic switch, reducing macrophage accumulation and necrotic core formation while increasing fibrous cap thickness, thereby stabilizing the atherosclerotic plaque. Created with BioRender.com.

The phenotypic plasticity of SMC is a major driver of atherosclerotic plaque complexity, yet the specific regulatory networks governing their transition to pathogenic, pro-inflammatory states have remained elusive [21]. In this study, we integrated single-cell trajectory analysis with gene regulatory network modeling to demonstrate that SMCs transdifferentiate into a distinct, pro-inflammatory macrophage-like population (macrophage 4) through an intermediate “stem–endothelial–monocyte” cell state. We identified IRF7 not merely as a passive marker, but as a master transcriptional regulator required for the acquisition and maintenance of this specific pathogenic identity. Crucially, we provide direct *in vivo* evidence that SMC-specific silencing of *Irf7* effectively blocks this transdifferentiation trajectory, leading to reduced plaque burden, diminished inflammation, and enhanced fibrous cap stability in hyperlipidemic mice. Furthermore, our analysis of human transcriptomic datasets validates the clinical translational potential of these findings, linking elevated IRF7 expression to plaque instability and IPH. Collectively, our data unveil a novel mechanism of maladaptive SMC plasticity and propose the targeted inhibition of IRF7 in the vascular wall as a potent strategy to stabilize vulnerable plaques (Fig. 8).

Our study adds to the growing body of evidence challenging the traditional binary view of SMC plasticity (contractile vs. synthetic) [22]. Consistent with the single-cell genomic atlas reported by Pan *et al*. [14], we identified the “stem–endothelial–monocyte” cell state as a pivotal intermediate hub. This confirms that SMC dedifferentiation is not a stochastic jump to a new identity, but a stepwise trajectory through a multipotent, stem-like progenitor state. This observation resonates with the emerging “athero-oncology” perspective [23], which conceptualizes atherosclerosis as a SMC–driven, tumor-like disease characterized by extensive dedifferentiation and clonal expansion [24]. However, the ultimate fate of these dedifferentiated cells has been the subject of recent debate. While some studies suggest that SMCs predominantly differentiate into protective “fibromyocytes” and contribute minimally to the macrophage pool, our lineage-tracing analysis aligns with Pan *et al*. and others in identifying a substantial population of SMC-derived macrophage-like cells [20]. Crucially, our study refines this understanding by resolving the functional heterogeneity within this transdifferentiated population. We demonstrate that SMC-derived macrophage-like cells bifurcate into distinct subsets: a less inflammatory state (macrophage 3) and a highly pro-inflammatory state (macrophage 4). By isolating the specific trajectory of macrophage 4, we provide a higher-resolution map of the “pathogenic” arm of SMC plasticity.

Our identification of IRF7 as a driver of plaque vulnerability aligns with a broader emerging paradigm implicating the interferon regulatory factor (IRF) family in the orchestration of atherosclerotic inflammation. Previous studies have extensively characterized IRF5 as a master regulator of macrophage polarization toward a pro-inflammatory phenotype. Seneviratne *et al*. demonstrated that IRF5 promotes necrotic core formation by impairing macrophage efferocytosis and driving CD11c^+^ cell accumulation [25, 26]. Consistent with this, myeloid-specific deletion of Irf5 has been shown to stabilize atherosclerotic lesions by resolving inflammation, and distinct IRF5-dependent macrophage populations have been directly linked to plaque rupture in human patients [25–27]. Similarly, recent single-cell transcriptomic analyses have highlighted IRF8 as another pivotal regulator governing macrophage burden and inflammatory signaling within the lesion [28]. Besides, absence of IRF1 protects against atherosclerosis in *ApoE^−/−^* mice [29]. Our study extends this concept by revealing that SMC-derived cells acquiring a macrophage-like identity also co-opt an IRF-driven regulatory program—specifically IRF7—to fuel plaque instability. This suggests a conserved “IRF-centric” mechanism of pathogenicity that operates across both bone marrow-derived myeloid cells and transdifferentiated SMCs.

Several factors have been identified to take parts in the phenotype switching of SMC in atherosclerosis [5, 30–37]. Our identification of IRF7 as a pathogenic driver in atherosclerosis presents an intriguing contrast to its previously reported roles in other vascular conditions. Prior studies by Huang *et al*. and Deng *et al*. established IRF7 as a protective factor in the contexts of mechanical carotid injury and pulmonary hypertension, respectively [11, 12]. In those models, IRF7 functioned primarily by inhibiting SMC proliferation, thereby limiting neointimal hyperplasia and vascular remodeling. However, our results in the *ApoE^−/−^* atherosclerosis model reveal a distinct, deleterious function for SMC-derived IRF7. This discrepancy likely reflects the fundamental differences in disease etiology: while restenosis is driven largely by rapid, maladaptive proliferation, atherosclerosis is orchestrated by chronic lipid accumulation and inflammation. In the lipid-rich plaque microenvironment, our data suggest that IRF7 does not merely regulate cell cycle progression, but rather orchestrates a transcriptional program driving transdifferentiation into a pro-inflammatory macrophage-like state. This finding aligns with Senatus *et al*., who reported that IRF7 expression in macrophages promotes inflammation and impairs cholesterol efflux in diabetic atherosclerosis [13]. Thus, IRF7 appears to act as a “double-edged sword” in the vasculature: potentially stabilizing the vessel wall in response to acute mechanical stress but fueling maladaptive phenotypic switching and plaque instability under chronic hyperlipidemic stress.

The discrepancy between the protective role of IRF7 in neointimal hyperplasia and its pathogenic role in atherosclerosis may be mechanistically explained by its interaction with ATF3. Previous studies established that IRF7 physically interacts with ATF3 in SMCs, functioning as a transcriptional repressor that inhibits ATF3-mediated expression of PCNA and cell proliferation. While this repression is beneficial in the context of restenosis (where rapid proliferation is the driver), the role of ATF3 in lipid-driven atherosclerosis appears fundamentally different. A recent study by Nie *et al*. demonstrated that ATF3 deficiency significantly exacerbates atherosclerosis, identifying ATF3 as a critical protective factor that limits vascular senescence and inflammation [38, 39]. In this context, we hypothesize that the robust upregulation of IRF7 in the atherosclerotic plaque may lead to the “inappropriate” sequestration or inhibition of ATF3. If IRF7 continues to function as an ATF3 antagonist within the plaque microenvironment—as it does in injured arteries—it would effectively suppress the protective, anti-inflammatory, and anti-senescence functions of ATF3. This suggests a model where IRF7 exacerbates atherosclerosis not only by directly driving a pro-inflammatory macrophage-like fate but also by dismantling the endogenous protective brake provided by ATF3. Future studies exploring the protein–protein interaction between IRF7 and ATF3 specifically within the context of hyperlipidemia will be essential to validate this potential double-hit mechanism.

The translational relevance of our findings is underscored by the robust upregulation of IRF7 in unstable and advanced human atherosclerotic plaques. Current standard-of-care therapies, such as statins and proprotein convertase subtilisin/kexin type 9 (PCSK9) inhibitors, are highly effective at lowering systemic lipids but do not fully abrogate the risk of plaque rupture, leaving a significant “residual inflammatory risk” [40, 41]. Our data suggest that IRF7 drives the precise features associated with plaque vulnerability: a large necrotic core, thinned fibrous cap, and intense local inflammation. By identifying IRF7 as the master regulator of the pro-inflammatory SMC transition, we highlight a novel therapeutic target located within the vessel wall itself, distinct from systemic lipid metabolism. Strategies capable of inhibiting IRF7 function—or its downstream effectors in the SMC lineage—could potentially prevent the deviation of SMCs into pathogenic macrophage-like cells. Such an approach could offer a complementary avenue to lipid-lowering therapies, specifically aimed at promoting structural plaque stability and preventing catastrophic clinical events [42].

Despite these promising findings, our study has several limitations that warrant consideration and outline important avenues for future investigation.

First, regarding our loss-of-function model, while our AAV-mediated shRNA strategy effectively reduced IRF7 protein levels and significantly ameliorated plaque pathology, it does not achieve the complete and permanent ablation provided by a germline genetic KO. Residual IRF7 expression in our *Irf7* KD mice may have underestimated the full pathogenic potential of this transcription factor. Future studies utilizing smooth muscle-specific conditional KO mice (e.g. Myh11-CreERT2; *Irf7*^*fl/fl*^) would provide more definitive evidence and allow for the precise temporal dissection of IRF7 function at different stages of atherogenesis.

Second, our mechanistic mapping of the IRF7 gene regulatory network relies primarily on computational inference from single-cell transcriptomic data. While the *in silico* perturbation analysis (CellOracle) strongly predicts a causal role for IRF7 in orchestrating the macrophage-like trajectory, direct physical validation of these regulatory interactions remains to be performed. We hypothesize that IRF7 may cooperate with other activator protein 1 (AP-1) superfamily members (e.g. ATF3) to remodel the SMC chromatin landscape. Future investigations employing ChIP-seq in primary SMCs will be essential to map the direct genomic binding sites of IRF7 and elucidate the specific “pioneer” factors it recruits during phenotypic switching.

Third, while we confirmed the reduction of the general macrophage marker CD68 and the specific SMC-lineage marker CD200 upon *Irf7* KD, our assessment of the inflammatory milieu relied largely on transcriptomic signatures. We did not explicitly quantify the protein levels of secreted cytokines (e.g. IL-1β, TNF-α, IL-6) within the plaque tissue. Given the strong correlation we observed between IRF7 and the NLR family pyrin domain containing 3 (NLRP3) inflammasome pathway in human plaques, future work should focus on determining whether SMC-specific IRF7 directly primes the inflammasome machinery, thereby identifying a potential “druggable” axis to block plaque inflammation without compromising systemic immunity.

## Conclusion

In conclusion, this study delineates a previously unrecognized pathogenic axis in atherosclerosis, wherein SMCs transdifferentiate into a specific pro-inflammatory macrophage-like state driven by the transcription factor IRF7. We demonstrate that this molecular switch is not merely a marker of disease progression but a functional driver of plaque instability. By identifying the specific regulatory network governing this transition, our findings provide a roadmap for developing precision therapies that target the root cause of vascular inflammation. Interventions capable of inhibiting IRF7 hold the potential to stabilize vulnerable plaques and reduce the residual risk of cardiovascular events in patients with advanced atherosclerosis (Fig. 8).

## Supplementary Material

pbaf039_Supplemental_File
