# Supplementary material for: IRF7 orchestrates maladaptive smooth muscle cell phenotype switching in atherosclerosis
Source: Precis Clin Med. 2025 Dec 27;9(1):pbaf039. doi: 10.1093/pcmedi/pbaf039 (PMC12859258; doi:10.1093/pcmedi/pbaf039)
Supplement: pbaf039_Supplemental_File [file pbaf039_supplemental_file.docx]

**
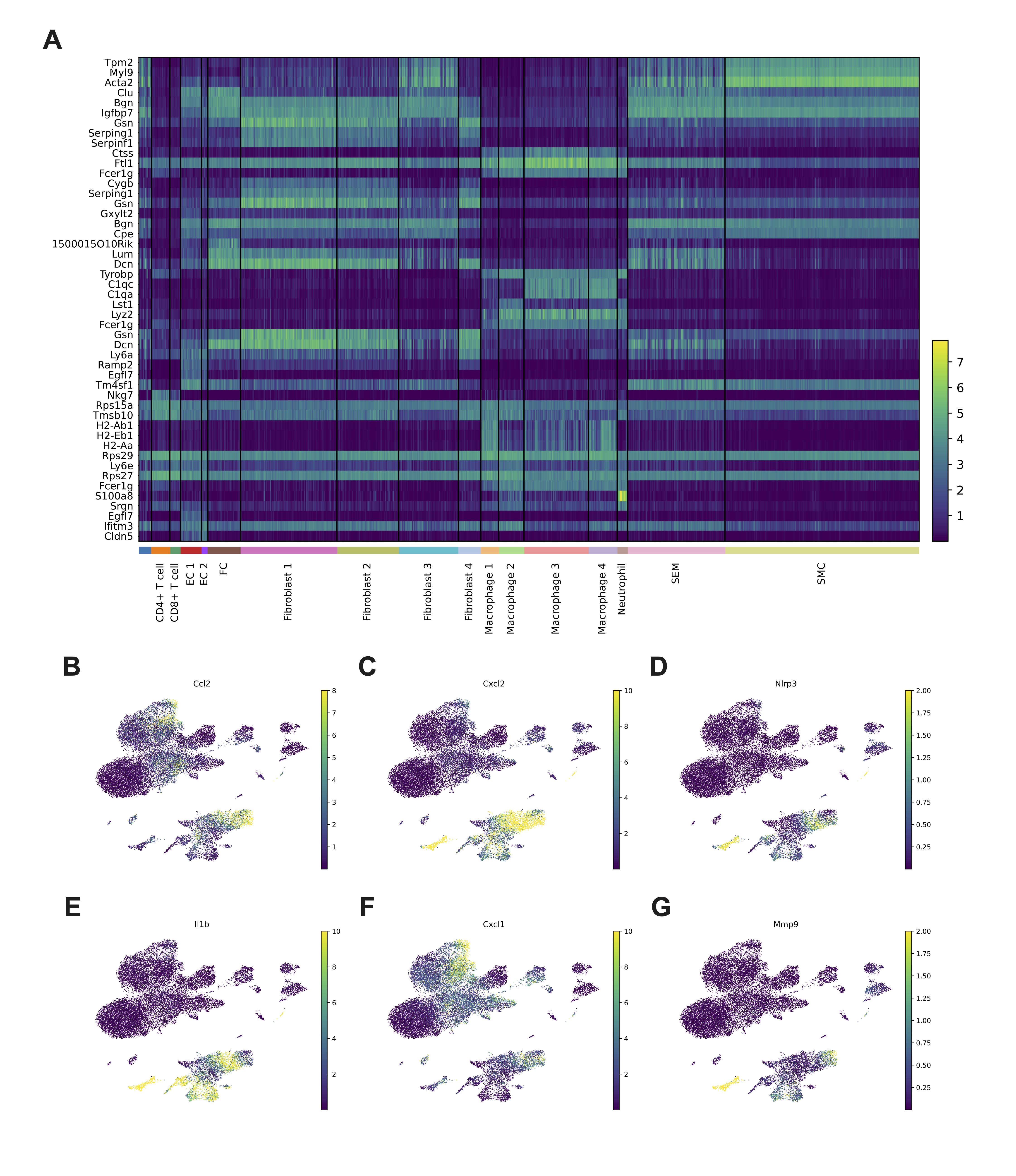
Supplementary materials**

**Supplementary Figure 1. Transcriptional characterization of cell clusters and inflammatory signatures in the single-cell dataset.** (A) Heatmap displaying the top differentially expressed genes (DEGs) defining each of the 16 cell clusters identified in the scRNA-seq analysis. Columns represent individual cells, and rows represent genes. The scale bar indicates relative expression levels. (B–G) Feature plots visualizing the expression of key pro-inflammatory genes on the UMAP embedding. High expression of these inflammatory mediators is specifically enriched within the Macrophage 4 cluster, distinguishing it from the less inflammatory Macrophage 3 population.


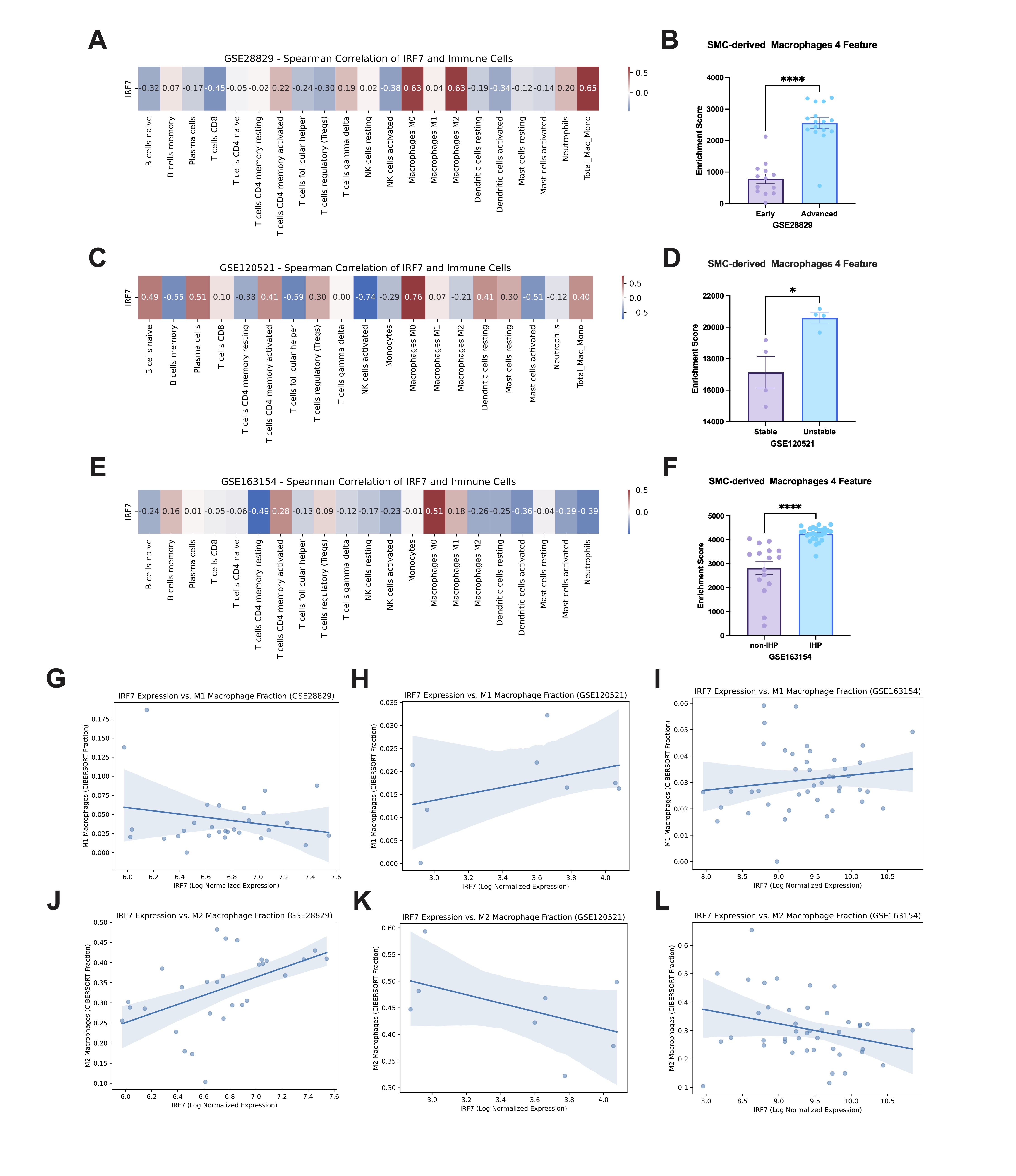


**Supplementary Figure 2. Immune infiltration and Macrophage 4 signature enrichment in human atherosclerotic plaques.** (A, C, E) CIBERSORT immune deconvolution analysis showing the estimated fractions of total Macrophages and Monocytes in three independent human datasets. Severe plaque phenotypes exhibit significantly higher macrophage burden. (B, D, F) Single-sample Gene Set Enrichment Analysis (ssGSEA) of the "Macrophage 4" gene signature (top 100 DEGs from scRNA-seq). The pro-inflammatory Macrophage 4 signature is significantly enriched in Unstable, IPH, and Advanced plaques compared to their respective controls. (G–L) Spearman correlation analysis between IRF7 expression and CIBERSORT-estimated fractions of M1-like (pro-inflammatory) and M2-like (anti-inflammatory) macrophages. Data are presented as mean SEM. **P* < 0.05, ***P* < 0.01, ****P* < 0.001.


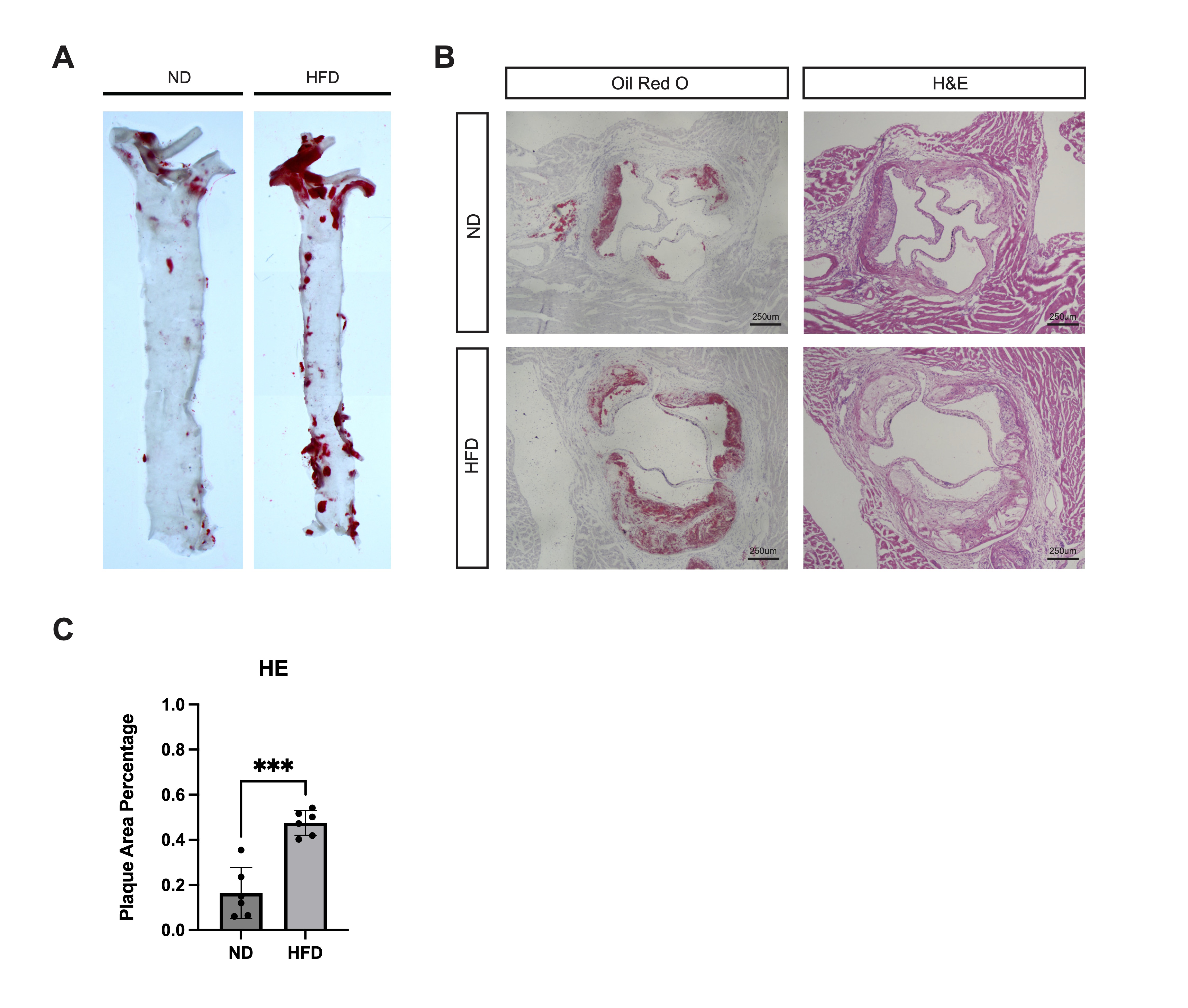


**Supplementary Figure 3. Validation of the ApoE^-/-^ high-fat diet atherosclerosis model.** (A) Representative en face Oil Red O staining of aortas from ApoE^-/-^ mice fed a Normal Diet (ND) or Western Diet (HFD) for 20 weeks. HFD feeding induces extensive atherosclerotic lesion formation. (B) Representative cross-sectional images of the aortic root stained with Hematoxylin & Eosin (H&E) and Oil Red O, and (C) the quantification of plaque size. The HFD group exhibits significant plaque burden and lipid accumulation compared to the plaque-free ND group. Data are presented as mean SEM. **P* < 0.05, ***P* < 0.01, ****P* < 0.001.


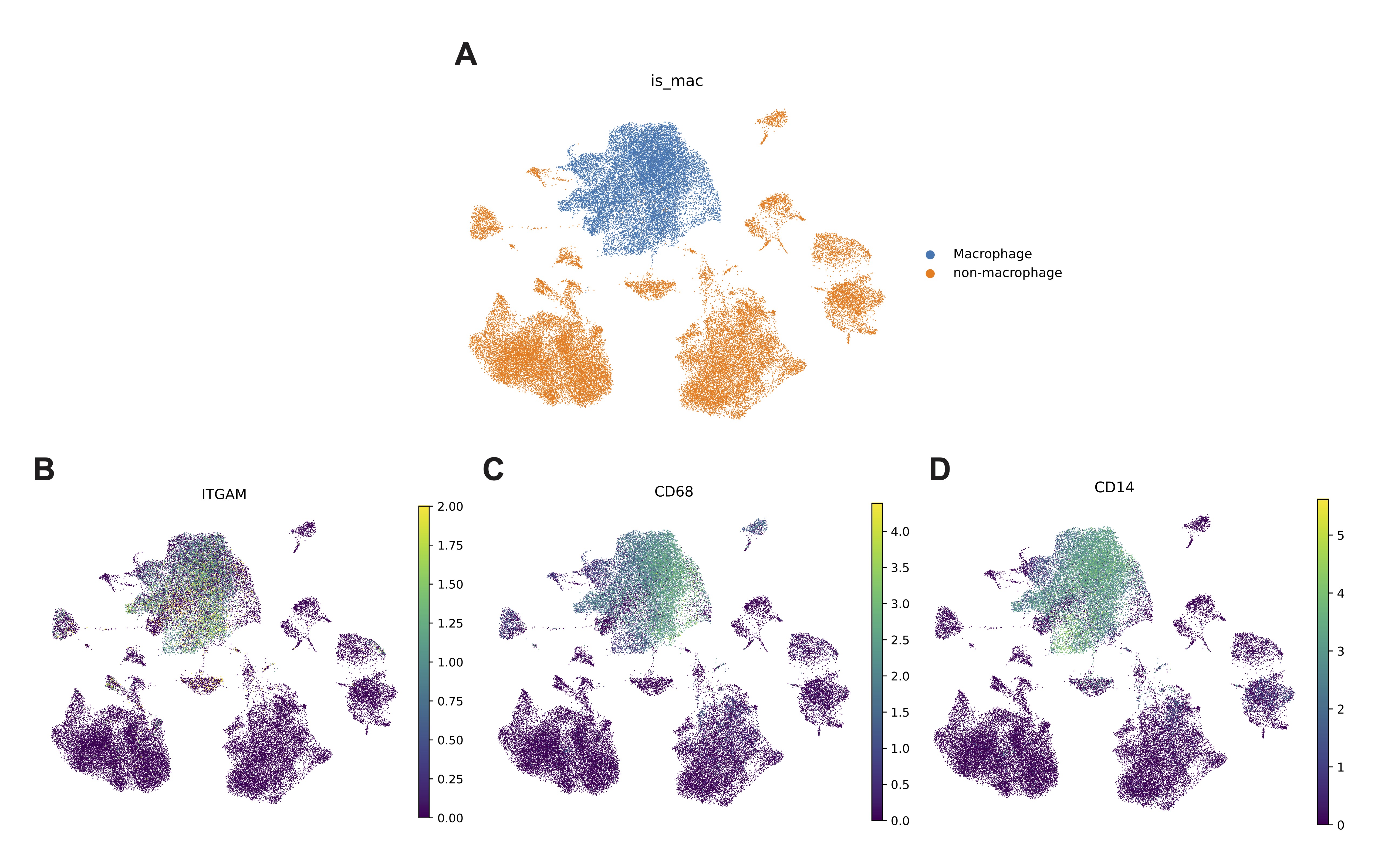


**Supplementary Figure 4 Identification and gating of macrophages in the human carotid atherosclerosis single-cell dataset.** (A) UMAP projection of all cells integrated from the human carotid atherosclerosis dataset (n=12 donors). The cluster identified as "Macrophages" based on canonical marker expression is highlighted in blue versus non-macrophage clusters. (B–D) Feature plots validating the macrophage identity of the selected cluster. The selected population shows high and specific expression of the canonical myeloid markers (B) ITGAM (CD11b), (C) CD68, and (D) CD14. This gated population was used for the downstream sub-clustering and lineage analysis shown in Figure 5.

**.**
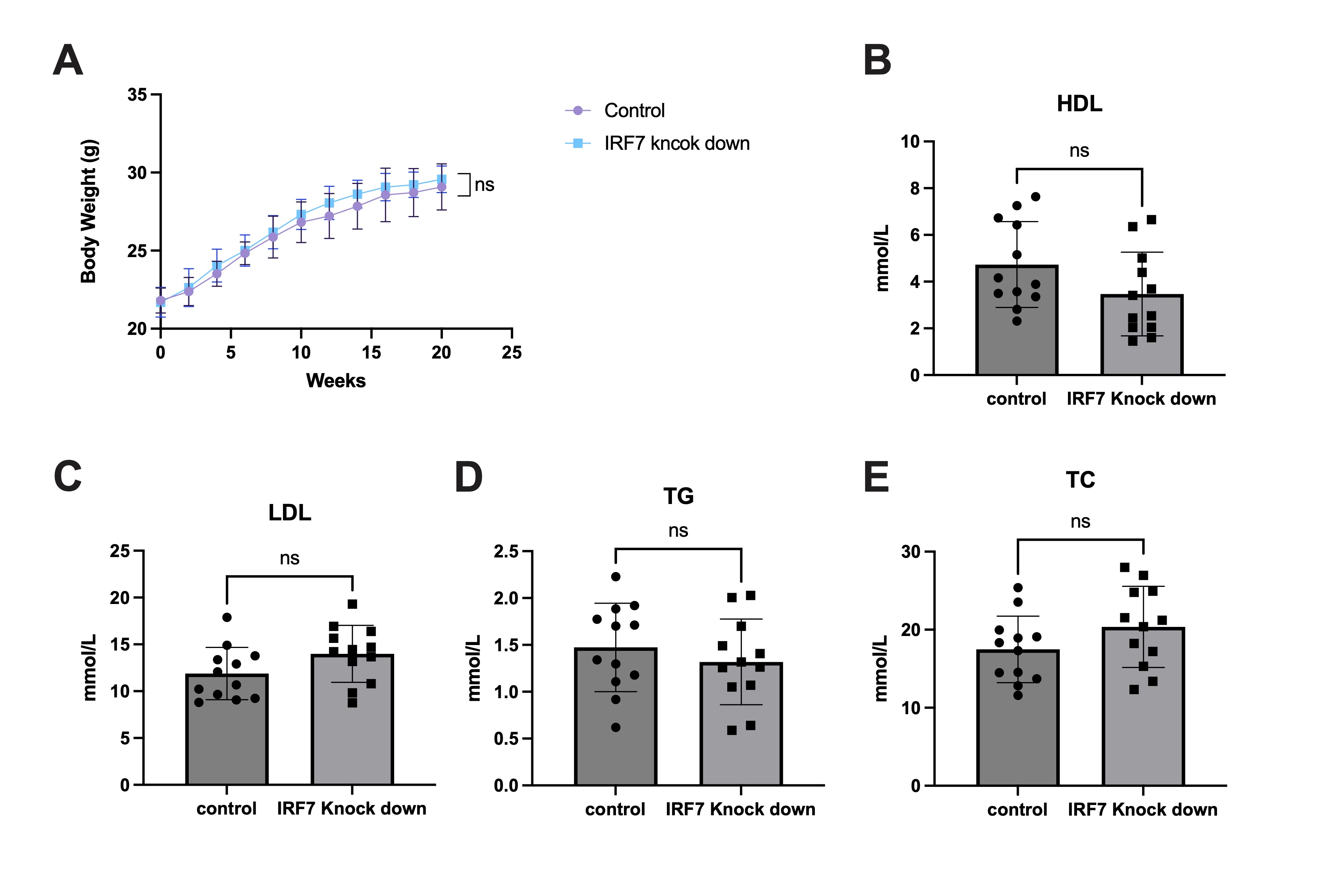


**Supplementary Figure 5. Metabolic parameters of ApoE^-/-^ mice following SMC-specific Irf7 knockdown.** (A–E) Quantitative analysis of metabolic parameters in Control (AAV-Scramble) and IRF7 KD (AAV-shIRF7) mice after 20 weeks of Western Diet feeding. No significant differences were observed between groups in (A) Body Weight, (B) HDL-Cholesterol levels, (C) LDL-Cholesterol, (D) Triglycerides, or (E) Total Cholesterol. Data are presented as mean SEM. **P* < 0.05, ***P* < 0.01, ****P* < 0.001.

**Supplementary Table 1. Antibody used in western blot.**

| Antibody | Brand | Lot | Dilution ratio |
| --- | --- | --- | --- |
| Rabbit anti-IRF7 Polyclonal antibody | Proteintech | 22392-1-AP | 1:20000 |
| Rabbit Recombinant Multiclonal CD68 antibody | Abcam | ab303566 | 1:2000 |
| Rabbit Recombinant Anti-GAPDH antibody | Servicebio | GB15004-100 | 1:20000 |
| HRP-conjugated Goat Anti-Rabbit IgG(H+L) | Proteintech | SA00001-2 | 1:20000 |

**Supplementary Table 2. Antibody used in IHC, mIHC, and IF.**

| Antibody | Brand | Lot | Dilution ratio |
| --- | --- | --- | --- |
| Mouse anti-IRF7 monoclonal antibody | Santa Cruz | sc-74471 | 1:25 |
| Rabbit CD68 Recombinant monoclonal antibody | Proteintech | 83014-5-RR | 1:1000 |
| Mouse Anti-α-Smooth Muscle Actin (ACTA2) monoclonal Antibody | Merck | A2547 | 1:500 |
| Rabbit CD200 monoclonal antibody | Abcam | ab314662 | 1:500 |
| Goat anti-Mouse IgG (H+L) Cross-Adsorbed Secondary Antibody, Alexa Fluor™ 488 | Thermo Fisher | A-11001 | 1:500 |
| Goat anti-Rabbit IgG (H+L) Cross-Adsorbed Secondary Antibody, Alexa Fluor™ 594 | Thermo Fisher | A-11012 | 1:500 |
| S-vision IHC general secondary antibody | Servicebio | G1303 | Ready to use |

**Bioinformation methods**

**Data Processing, Quality Control, and Cell Type Annotation**

**Data Preprocessing and Doublet Removal**

Raw gene expression matrices were imported into Python using the Scanpy toolkit. To ensure high-quality data input, initial filtering was performed to remove genes expressed in fewer than 10 cells. For each sample, highly variable genes (n=2,000) were selected using the Seurat v3 flavor for initial dimensionality reduction. Doublet detection was performed using SOLO (scvi-tools), a deep learning-based doublet detection method. A variational autoencoder (VAE) model was trained on the data, and cells predicted as doublets with a high confidence differential were excluded from downstream analysis.

**Quality Control (QC)**

Following doublet removal, rigorous quality control metrics were calculated. Cells were filtered based on three criteria to remove low-quality cells and potential debris: Library Size: Cells with an anomalously high number of detected genes were removed (upper limit set at the 98th percentile). Mitochondrial Content: The percentage of mitochondrial gene counts was calculated, and cells exceeding the 98th percentile for mitochondrial content were excluded to minimize the inclusion of dead or dying cells. Minimum Gene Count: After merging samples, a final filter was applied to retain only genes expressed in at least 100 cells across the entire dataset.

**Data Integration and Normalization**

To correct for batch effects arising from different sequencing runs and experimental conditions (Time Point and Lineage Origin), the probabilistic model scVI (single-cell Variational Inference) was employed. The raw count data were used as input for the scVI model, with order (sequencing batch), and week (0, 8, 16, 26 weeks) treated as categorical covariates. Continuous covariates included the percentage of mitochondrial counts and total counts. The model was trained to learn a low-dimensional latent representation (X_scVI) of the data, which was used for all subsequent neighborhood graph construction and dimensionality reduction. Denoised and normalized expression values were also generated by the scVI model for differential expression analysis.

**Clustering and Dimensionality Reduction**

A k-nearest neighbor (k-NN) graph was constructed using the scVI latent representation. Uniform Manifold Approximation and Projection (UMAP) was performed to visualize the data in two dimensions. Unsupervised clustering was carried out using the Leiden algorithm with a resolution of 0.83, identifying distinct cell communities based on transcriptional profiles.

**Cell Type Annotation**

Cluster-specific marker genes were identified using the rank_genes_groups function (Wilcoxon rank-sum test). Clusters were annotated based on the expression of canonical marker genes and aligned with the cell types described in the source dataset (Pan et al.). The resulting annotation identified 16 distinct cell types/states, including SMC (Smooth Muscle Cells), SEM (Stem cell, Endothelial cell, Monocyte-like), FC (Fibrochondrocytes), various fibroblast subtypes (Fibroblast 1–4), macrophage subsets (Macrophage 1–4), endothelial cells (EC 1–2), T cells (CD4+ and CD8+), and neutrophils.

**Differential Expression and Functional Enrichment Analysis**

Differential Expression Analysis To identify marker genes and transcriptomic signatures specific to each cell cluster, differential expression (DE) analysis was performed. Leveraging the probabilistic framework of the scVI model, differential expression was assessed to estimate the magnitude of change between groups. Genes with a positive log-fold change (lfc_mean > 0) were prioritized to identify upregulated markers characteristic of specific cell states (e.g., Macrophage subpopulations).

Visualization of Gene Expression Gene expression patterns were visualized using the scVI-normalized expression values (layer='scvi_normalized') to account for technical variations and library size differences. Feature plots were generated to display the spatial distribution of key inflammatory markers (e.g., Ccl2, Cxcl2, Nlrp3) on the UMAP embedding. Additionally, heatmaps were constructed to visualize the expression profiles of selected marker genes across defined cell types, with expression values log-transformed for clarity.

Functional Enrichment Analysis To elucidate the biological functions associated with the differentially expressed genes, Gene Ontology (GO) enrichment analysis was performed using the gseapy Python library. The list of upregulated genes identified from the DE analysis was tested against the GO_Biological_Process_2023 gene set library. To ensure statistical rigor, the analysis used the entire set of detected genes in the dataset as the background reference. Significant pathways were filtered based on an adjusted P-value < 0.05. The top enriched biological processes were ranked by statistical significance (-log10 adjusted P-value) and visualized using bar plots.

**Transcription Factor Network Analysis**

Single-Cell Regulatory Network Inference (SCENIC) To identify potential master transcriptional regulators driving the transition between specific SMC-derived states, we performed gene regulatory network (GRN) analysis using the pySCENIC pipeline (Python implementation of SCENIC). This analysis was specifically focused on the SMC-derived macrophage populations. We subsetted the data to include only cells identified as SMC-lineage (SMC_origin == 'Yes') and belonging to the Macrophage 3 and Macrophage 4 clusters.

Network Inference and Motif Enrichment The analysis followed the standard three-step workflow:

Co-expression Network Inference: First, a co-expression network was inferred from the raw gene count matrix using the GRNBoost2 algorithm. A list of curated mouse transcription factors (allTFs_mm.txt) was used as input to predict potential regulatory interactions (adjacencies).

Regulon Prediction (Cis-regulatory Motif Analysis): To filter false positives and identify direct targets, the co-expression modules were pruned using cis-regulatory motif analysis. We utilized the mm9 genomic ranking databases to search for enriched motifs within the regulatory regions of the co-expressed genes. Only modules with significant motif enrichment were retained as "regulons" (a transcription factor and its direct target genes).

Regulon Activity Scoring (AUCell): Finally, the cellular activity of each regulon was quantified using AUCell (Area Under the Curve), which calculates an activity score for each regulon in each individual cell based on the enrichment of its target genes. This allowed for the identification of differentially active transcription factors, including IRF7, between the Macrophage 3 and Macrophage 4 subpopulations.

**Gene Regulatory Network Modeling and In Silico Perturbation**

**Trajectory Inference and Pseudotime Analysis**

To establish the developmental trajectory within the SMC-derived macrophage lineage, the CellOracle library was utilized. The dataset was subsetted to include only SMC-lineage cells (SMC_origin == 'Yes') belonging to the specific macrophage clusters. A diffusion map was calculated to estimate the differentiation trajectory. Root cells were identified based on the UMAP coordinates corresponding to the earliest developmental state, and pseudotime values were assigned to each lineage to define the directionality of cell state transitions.

**Network Inference and Centrality Scoring**

A cell-type-specific gene regulatory network (GRN) was constructed to identify key transcription factors (TFs) driving cell identity.

Base Network: A base GRN structure was initialized using the compiled mouse scATAC-seq atlas provided within CellOracle, serving as a prior for potential regulatory interactions.

Imputation: To address gene dropout events common in scRNA-seq data, k-nearest neighbor (kNN) imputation was performed. The number of principal components (n_comps) and the k-value (k) were determined automatically based on the dataset structure and cell number.

Link Construction: Regularized regression models (Bayesian Ridge) were trained to predict gene expression based on TF expression (alpha=10). The resulting network links were filtered for statistical significance (P < 0.001) and strength (top 10,000 links based on absolute coefficient values).

Network Scoring: To identify pivotal regulators, network centrality metrics were calculated. Eigenvector centrality scores were compared between clusters (Macrophage 3 vs. Macrophage 4) to highlight TFs with differential regulatory influence, identifying IRF7 as a top-ranking regulator.

**In Silico Perturbation Analysis**

To predict the phenotypic consequences of Irf7 loss on cell identity, an in silico knockout simulation was performed using the CellOracle simulation module followed by the protocol in https://morris-lab.github.io/CellOracle.documentation/.

Visualization: The predicted shift in cell identity was visualized on the existing UMAP embedding using quiver plots (vector fields showing directionality) and grid-based simulation flow, illustrating how the loss of Irf7 alters the developmental trajectory of SMC-derived macrophages.

**Re-analysis of Human Single-Cell Data**

Publicly available scRNA-seq data from human carotid atherosclerotic plaques (Bashore et al., 2024) was obtained. Macrophages were computationally gated based on the expression of ITGAM, CD68, and CD14. To identify SMC-derived macrophage-like cells, we utilized CD200 as a lineage-specific marker. Differential expression analysis of IRF7 and inflammatory cytokines (IL1B, IL6, NLRP3) was performed between symptomatic and asymptomatic plaque groups using the Wilcoxon rank-sum test. Dimensionality reduction and visualization were performed using UMAP.

**Bulk RNA-Seq Data Analysis**

**Data Processing and Differential Expression**

Raw expression matrices or normalized series matrix files for datasets GSE28829, GSE163154, and GSE120521 were downloaded from the GEO database. For microarray data, probe IDs were mapped to gene symbols using the corresponding platform annotation files. In cases where multiple probes mapped to the same gene, the mean expression value was calculated. Expression values were log-transformed to ensure normal distribution prior to analysis. The differential expression of IRF7 between pathological groups (Stable vs. Unstable; IPH vs. Non-IPH; Early vs. Advanced) was evaluated using an unpaired Student’s t-test or Mann-Whitney U test, depending on data normality.

**Diagnostic Value Evaluation**

To assess the potential of IRF7 as a diagnostic biomarker for distinguishing critical atherosclerotic phenotypes, Receiver Operating Characteristic (ROC) curve analysis was performed. The Area Under the Curve (AUC) was calculated using the pROC package (or equivalent Python library like scikit-learn) to quantify the sensitivity and specificity of IRF7 expression in predicting plaque stability and progression.

**Immune Infiltration Analysis (CIBERSORT)**

To investigate the relationship between IRF7 expression and the immune microenvironment within atherosclerotic plaques, the CIBERSORT algorithm was applied to the datasets. The LM22 signature matrix, which defines 22 distinct immune cell subtypes, was used as the reference. Gene expression profiles were deconvoluted to estimate the relative fractions of infiltrating immune cells. Samples with a CIBERSORT P-value < 0.05 were retained for downstream analysis to ensure the reliability of the deconvolution results.

**Correlation Analysis**

Spearman’s rank correlation analysis was performed to evaluate the association between IRF7 expression levels and the abundance of specific infiltrating immune cells (e.g., Macrophages M1/M2, T cells) and smooth muscle cell markers. Correlation heatmaps and scatter plots were generated to visualize these relationships.
